# Isatuximab Monotherapy for Desensitization in Highly Sensitized Patients Awaiting Kidney Transplant

**DOI:** 10.1681/ASN.0000000000000287

**Published:** 2023-12-26

**Authors:** Flavio Vincenti, Oriol Bestard, Amarpali Brar, Josep M. Cruzado, Daniel Seron, A. Osama Gaber, Nicole Ali, Anat R. Tambur, Helen Lee, Giovanni Abbadessa, Jo-Anne Paul, Markus Dudek, Ruby J. Siegel, Alba Torija, Dorothée Semiond, Lucie Lépine, Nils Ternes, Robert A. Montgomery, Mark Stegall

**Affiliations:** 1 Departments of Medicine and Surgery, University of California San Francisco, San Francisco, California; 2 Department of Nephrology and Kidney Transplantation, University Hospital Vall d’Hebron, Barcelona, Spain; 3 Nephrology and Kidney Transplantation Laboratory, Vall d’Hebron Research Institute (VHIR), Barcelona, Spain; 4 Department of Medicine, University of California San Francisco, San Francisco, California; 5 Department of Nephrology, Hospital Universitari de Bellvitge, University of Barcelona, Barcelona, Spain; 6 Bellvitge Biomedical Research Institute (IDIBELL), L'Hospitalet de Llobregat, Barcelona, Spain; 7 Department of Surgery, Houston Methodist Hospital, Houston, Texas; 8 Department of Surgery, Transplant Institute, New York University Langone Health, New York, New York; 9 Department of Surgery, Northwestern University Feinberg School of Medicine, Chicago, Illinois; 10 Sanofi, Cambridge, Massachusetts; 11 Sanofi, Bridgewater, New Jersey; 12 Sanofi R&D, Frankfurt, Germany; 13 Sanofi R&D, Chilly-Mazarin, France; 14 Department of Surgery, Mayo Clinic Rochester, Rochester, Minnesota

**Keywords:** clinical trial, kidney transplantation, pharmacokinetics, rejection, transplantation

## Abstract

**Significance Statement:**

There is no standardized desensitization regimen for kidney transplant candidates. CD38, expressed by plasma cells, could be targeted for desensitization to deplete plasma cells producing alloantibodies and donor-specific antibodies. Few studies and case reports are available regarding the use of CD38 antibodies for desensitization in patients awaiting kidney transplant. This study shows that isatuximab, a CD38-targeting therapy, was well tolerated in kidney transplant candidates, with a durable decrease in anti-HLA antibodies and partial desensitization activity. The short treatment period and long follow-up of this study allowed for the understanding of the mechanism and timing for any antibody rebound. Isatuximab could be further investigated as an option for adjunct therapy to existing desensitization for patients on the kidney transplant waitlist.

**Background:**

Patients with calculated panel reactive antibody (cPRA) ≥80.00%, particularly those with cPRA ≥99.90%, are considered highly sensitized and underserved by the Kidney Allocation System. Desensitization removes circulating reactive antibodies and/or suppresses antibody production to increase the chances of a negative crossmatch. CD38 is expressed highly on plasma cells, thus is a potential target for desensitization.

**Methods:**

This was an open-label single-arm phase 1/2 study investigating the safety, pharmacokinetics, and preliminary efficacy of isatuximab in patients awaiting kidney transplantation. There were two cohorts, cohorts A and B, which enrolled cPRA ≥99.90% and 80.00% to <99.90%, respectively.

**Results:**

Twenty-three patients (12 cohort A, 11 cohort B) received isatuximab 10 mg/kg weekly for 4 weeks then every 2 weeks for 8 weeks. Isatuximab was well tolerated with pharmacokinetic and pharmacodynamic profiles that indicated similar exposure to multiple myeloma trials. It resulted in decreases in CD38^+^ plasmablasts, plasma cells, and NK cells and significant reductions in HLA-specific IgG-producing memory B cells. Overall response rate, on the basis of a predefined composite desensitization end point, was 83.3% and 81.8% in cohorts A and B. Most responders had decreases in anti-HLA antibodies that were maintained for 26 weeks after the last dose. Overall, cPRA values were minimally affected, however, with only 9/23 patients (39%) having cPRA decreases to target levels. By study cutoff (median follow-up of 68 weeks), six patients received transplant offers, of which four were accepted.

**Conclusions:**

In this open-label trial, isatuximab was well tolerated and resulted in a durable decrease in anti-HLA antibodies with partial desensitization activity.

**Clinical Trial registration number:**

NCT04294459.

## Visual Abstract

**Figure:**
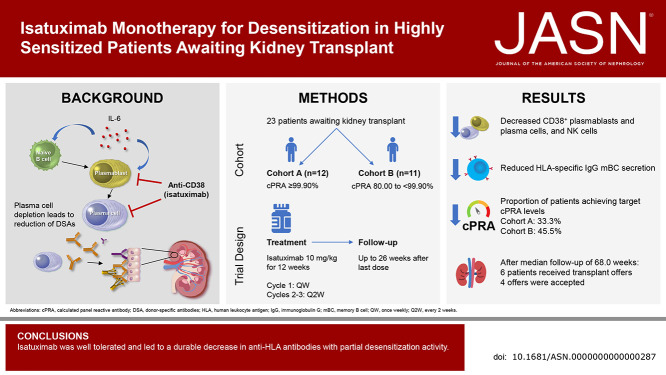

## Introduction

Patients may become sensitized to HLAs through pregnancy, after a blood product transfusion, or after solid organ transplantation.^1^ Although there is no standardized definition, highly sensitized patients can be regarded as patients with a calculated panel reactive antibody (cPRA) ≥80.00%.^2,3^ Only 6.5% of highly sensitized patients with cPRA ≥80.00% receive a compatible kidney transplant each year, and almost none of these patients have a cPRA ≥99.90%.^4^

The implementation of the kidney allocation system (KAS) in 2014 dramatically increased organ equity for highly sensitized patients and increased the likelihood of finding a compatible donor for transplantation. However, post-KAS implementation, candidates with cPRA ≥99.90% continue to have very low rates of kidney transplantation. These patients on average receive less than one organ offer per decade and have the greatest need for desensitization.^5^

The aim of desensitization is to remove circulating HLA-reactive antibodies and/or reduce or eliminate antibody production, thus increasing the chances of a negative crossmatch and of matching with a compatible donor, reducing time on dialysis, and improving clinical outcome.^6^ However, there is no standard desensitization regimen, and most regimens involve off-label plasmapheresis, intravenous Ig, and anti-CD20 therapies, such as rituximab.^4^ Other trials have also investigated IL-6 targeting therapies, such as tocilizumab and clazakizumab, or proteasome inhibitors, such as bortezomib and carfilzomib.^1^ Imlifidase, a cysteine protease that cleaves all IgG subclasses, was recently approved by the European Medicines Agency for desensitization treatment of highly sensitized adult kidney transplant recipients with positive crossmatch against an available deceased donor.^7,8^ However, imlifidase lacks durability of effect, requires repeated dosing that would not be feasible due to antidrug antibody (ADA) response, and leaves patients still susceptible to antibody-mediated rejection (ABMR) due to antibody rebound.^7^

CD38 is a commonly found ectoenzyme on plasma cells and multiple myeloma cells.^9^ Alloantibody-producing plasma cells express CD38 at a higher level than other CD38^+^ hematopoietic cells.^9^ There is thus a rationale for depleting plasma cells producing alloantibodies or donor-specific antibodies (DSAs) for desensitization in kidney transplant patients with CD38-targeting antibodies. Few studies are ongoing, and few clinical case reports are available regarding the use of anti-CD38 antibodies, which have demonstrated various levels of DSA reduction, for desensitization in patients awaiting transplantation.^10–14^

Isatuximab is an anti-CD38 monoclonal antibody approved in combination with pomalidomide and dexamethasone, and in combination with carfilzomib and dexamethasone, for the treatment of patients with relapsed/refractory multiple myeloma.^15^ Isatuximab binding to CD38 triggers a number of Fc-dependent mechanisms—antibody-dependent cellular cytotoxicity, complement dependent cytotoxicity, and antibody-directed cellular phagocytosis—and direct apoptosis.^16–18^ Isatuximab has demonstrated the induction of apoptosis in primary cells from bone marrow aspirates of patients with multiple myeloma.^16^ It is hypothesized that isatuximab can target long-lived plasma cells, depleting the production source of alloantibodies and DSAs, leading to their sustained removal.

In this phase 1/2 study report (NCT04294459), we describe the safety, pharmacokinetics (PK), and preliminary efficacy of isatuximab in patients awaiting kidney transplantation. The study included two cohorts on the basis of patient's baseline (BL) cPRA. Cohorts A and B enrolled patients with cPRA ≥99.90% and cPRA 80.00% to <99.90%, respectively, where the former represents a sample of a population particularly poorly served by the current KAS.

## Methods

This was an open-label, single-arm, phase 1/2 study conducted at six centers in the United States and Spain between June 18, 2020, and May 2, 2022. The study had a screening period of up to 28 days, a treatment period of up to 12 weeks, a site-visit follow-up of up to 26 weeks after treatment had stopped, and an extended follow-up *via* telephone every 90 days until study cut-off date, death, or loss to follow-up. Study cutoff was planned at 26 weeks after the last patient completed the treatment period or when the last ongoing patient was lost to follow-up, whichever was earlier. The primary objective of the phase 1 study was to characterize the safety and tolerability of isatuximab in kidney transplant candidates. The primary objective of the phase 2 study was to evaluate the preliminary efficacy of isatuximab in the desensitization of patients awaiting kidney transplantation.

### Isatuximab Treatment

Isatuximab was administered at a starting dose of 10 mg/kg every week for 4 weeks in cycle 1 and every 2 weeks for cycles 2 and 3. Patients underwent three cycles of treatment (a total of eight planned doses) spanning a 12-week period. Each cycle was 28 days.

### Study End Points

The primary end point for the phase 1 study was the proportion of patients with adverse events (AEs), serious AEs, and laboratory abnormalities. In phase 2, the primary end point was response rate (RR) which was a composite end point, as assessed by central laboratory. RR was defined as a proportion of patients meeting at least one of the three predefined desensitization efficacy criteria (see Supplemental Appendix 1 for details). Criterion 1 was the reduction of cPRA to target levels, where target cPRA was defined as cPRA that would result in at least doubling the theoretical likelihood of finding a compatible donor.^19^ Criterion 2 was the reduction of >2 antibody titers to reach target cPRA, and criterion 3 was the elimination of anti-HLA antibody as mean fluorescence intensity (MFI) reduced to <2000 for antibodies with BL MFI of ≥3000. Secondary end points included duration of response (DoR), number of anti-HLA antibody eliminated, change in cPRA and anti-HLA antibody levels, PK, and biomarkers. Safety assessments included AEs and serious AEs reported per Common Terminology Criteria for AEs v5.0, laboratory abnormalities, and incidence of ADAs against isatuximab.

### PK Analysis

Blood samples were collected mainly during cycle 1 at selected time points (predose, end of infusion [EOI], EOI+1 hour or EOI+4 hours, start of infusion+72 hours, and start of infusion+168 hours) and were used for isatuximab PK assessment by noncompartmental analysis. Analysis was performed with Phoenix WinNonlin version 8.2 (Pharsight). The Gyrolab Platform, a quantitative sandwich immunoassay using biotinylated anti-isatuximab antibodies bound by streptavidin beads within the Gyrolab Bioaffy CD microstructure for capture and Alexa Fluor 647-conjugated CD38 antibody for detection, was used to measure functional isatuximab (isatuximab with ≥1 site available to bind target) plasma levels, with a lower limit of quantitation of 5.0 *μ*g/ml.

### Anti-HLA Antibody Testing

Serum samples were frozen at −80°C for at least 10 minutes, then thawed at 2−4°C, and brought to room temperature for preparation. Aggregates were removed by centrifugation for 5 minutes at 7400×*g*. Serum was treated with Adsorb Out Beads (ADSORB, One Lambda) according to the manufacturer's instructions, and EDTA, 0.5 M pH 8.0±0.1, was added to serum in a 1:20 ratio (*e.g.*, 5 *μ*l EDTA to 95 *μ*l serum). Serum dilutions were performed in PBS (Beckman Coulter), and all assays were performed by one technologist. Consecutive samples from each patient were batched to minimize assay variability. Anti-HLA antibody testing was performed using LABScreen Single Antigen HLA class I (catalog LSA1A04, One Lambda) and LABScreen Single Antigen HLA class II (catalog LS2A01, One Lambda), and data were acquired on a LABScan 3D flow analyzer and analyzed in HLA Fusion 4.6 software.

### cPRA per Serial Dilutions

At BL, serum from each patient was tested neat and in serial doubling dilutions from 1:2 to 1:4096. Samples collected at day 1 of each treatment cycle and at site visit follow-up weeks 1, 5, 9, 13, 17, 21, and 25 were tested neat and at the relevant dilution for assessment of efficacy criterion 2. cPRA was calculated to two decimal points (*e.g.*, 99.99%) on the basis of the cPRA calculator developed by the Organ Procurement and Transplantation Network, with unacceptable antigens defined as those with MFI ≥2000.

### Antibody Titration Heat Maps to Compare BL to Follow-Up

Antibody results were compared for each patient for serum samples collected at BL versus site visit follow-up weeks 9 and 25 (or the closest dated alternate follow-up samples as available). HLA class I and class II panels were analyzed separately, and beads on each panel were sorted from high to low on the basis of the patient's BL titer strength for that bead. Following the BL titer sorted values, heat maps were produced in consecutive subsequent columns of a spreadsheet. For the BL and follow-up samples, MFIs for serum tested neat, 1:16, and 1:256 were compared. Conditional formatting was used to color code MFIs in strength categories.

### Biomarkers Analysis

Ig and immunophenotyping assays were performed by Covance Central Laboratory Services. B-cell panels, natural killer (NK) and natural killer T-cell panels, and Ig assays were performed.

B-cell panels were analyzed as follows—each specimen was incubated with Whole Blood Lysing Reagent and centrifuged afterward. White blood cells were then washed and prepared for immunophenotyping staining. Cells were incubated with Fc block working solution followed by incubation with CD38 FITC (Beckman Coulter), AHIgG1 FITC (Southern Biotech), CD24 PE (BioLegend), CD20 PerCP-Cy5.5 (BD Pharmingen), CD19 APC (BD Pharmingen), CD45 AF700 (BD Pharmingen), IgD V450 (BD Horizon), CD27 BV510 (BioLegend), and CD138 BV605 (BioLegend) in Brilliant Stain Buffer (BD Horizon). Finally, cells were fixed with 1% paraformaldehyde solution and acquired on the BD SORP FACSCanto II.

Memory B cells (mBC) were assessed both phenotypically and functionally. mBC phenotypes were assessed using peripheral blood mononuclear cells that were characterized by flow cytometry (Cytek Aurora CS) with the following markers: CD19 BV785 (BioLegend), CD20 BUV805 (Beckton Dickinson), CD27 PE-Cy7 (BioLegend), IgD SuperBright-436 (Thermo Fisher), CD24 BB515 (Beckton Dickinson), and CD38 R718 (Beckton Dickinson). Switched mBCs were defined as CD19^+^ CD20^+^ CD27^+^ IgD and analyzed according to the number of B cells/ml. For the evaluation of HLA-specific mBC function, mBCs were polyclonally stimulated as previously described in Luque *et al.*,^20^ seeded in an anti-IgG precoated FluoroSpot plate, and incubated overnight to release antibodies. HLA-sp mBC detection was performed with a HLA-sp B-cell FluoroSpot assay using different class I and class II fluorophore-conjugated HLA tetramers (Pure MHC; LLC, Oklahoma). All tested HLA specificities per patient and time point are listed in Supplemental Table 1. The results of the assay are reported as the number of IgG-secreting anti-HLA mBCs per 450,000 seeded cells as median and interquartile range.

For analysis of the NK and regulatory T-cell (Treg) cell panel, each specimen was washed with plain PBS without azide and the pellet was resuspended and incubated with *N*-hydroxysuccinimide solution. After washing, cells were incubated with Fc block working solution followed by incubation with CD38 FITC (Beckman Coulter), AHIgG1 FITC (Southern Biotech), CD25 PE (BD Pharmingen), CD127 PerCP-Cy5.5 (BioLegend), CD56 APC (BioLegend), *N*-hydroxysuccinimide Ester AF700 (Thermo Fisher), CD4 BV421 (BioLegend), CD8 BV510 (BD Horizon), and CD3 BV605 (BioLegend) in Brilliant Stain Buffer (BD Horizon). After washing, red blood cells were lysed with Whole Blood Lysing Reagent. Finally, cells were fixed with 1% paraformaldehyde solution and acquired on the BD FACSCanto II.

The bone marrow–residing HLA-specific plasma cell response analyses were performed as follows. Bone marrow aspirates were performed before first treatment and at the end of cycle 3 in three patients of the study. Bone marrow cells were isolated from bone marrow aspirates, seeded to an anti-IgG precoated FluoroSpot plate, and incubated for antibody release. HLA-sp IgG-secreting bone marrow plasma cells were detected and reported, as described previously for HLA-sp mBCs. HLA antigen specificities tested were randomly selected because of their antibody presence in the sera. See Supplemental Table 1 where all specificities tested in each patient are described.

### Statistical Analysis

Patients treated at the phase 2 dose during phase 1 were included in the efficacy analyses together with the phase 2 patients. RR (primary composite endpoint) was calculated as proportion of patients meeting at least one of the three predefined desensitization efficacy criteria, along with corresponding two-sided 95% confidence intervals (CIs) using the Clopper–Pearson method.

DoR was defined as the time from central laboratory sample collection date indicating response up to the central laboratory sample collection date when the patient was no longer meeting any response criterion (*i.e.*, nonresponder) or up to date of death due to any cause, whichever occurred first. DoR is summarized with the Kaplan–Meier method.

For pharmacodynamic analyses, the Wilcoxon signed-rank test was used to evaluate the significance of the change post-treatment as compared with the BL. To control for multiple testing, adjusted *P* values have been also calculated by using the Benjamini and Hochberg method.^21^

## Results

### Patients and BL Characteristics

**Figure 1:**
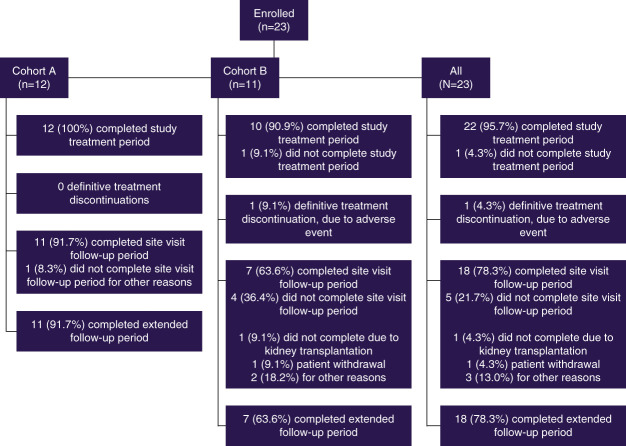
Patient disposition.

A total of 23 patients were enrolled in this study—12 in cohort A and 11 in cohort B—of which 22 completed the study treatment period and 18 completed the extended follow-up period until study cut-off date (Figure 1). The median follow-up of all treated participants was 68.0 weeks. One patient in cohort B discontinued treatment definitively on the basis of logistical reasons due to coronavirus disease 2019 positivity before the last planned dose.

The median age of patients was slightly higher in cohort A than cohort B (52.5 versus 48.0 years; Table 1). Patients in cohort A spent a median 6.0 years on the kidney transplant waitlist, while those in cohort B spent a median 3.6 years. Origins of renal insufficiency reported by investigators were also more varied in cohort B than in cohort A, with 50.0% of patients in cohort A due to prior transplant failure, compared with 36.4% in cohort B. Origins of renal insufficiency in cohort B also included urologic disorders (18.2%), hypertension (9.1%), and autosomal dominant polycystic kidney disease (9.1%). Most patients had one prior kidney transplant (58.3% cohort A, 54.5% cohort B), and over 90% of patients in both cohorts had prior sensitizing events at screening, mostly attributed to transplant and transfusion. All 23 patients were diagnosed with stage 5 CKD at the time of study entry, representing a patient population that is likely to require dialysis (22 of 23 patients were on dialysis at the time of study entry).

**Table 1:** Baseline characteristics

Characteristic	Cohort A (*n*=12)	Cohort B (*n*=11)	All (*N*=23)
Median age, yr (min–max)	52.5 (25–68)	48.0 (25–69)	52.0 (25–69)
Sex, *n* (%)			
Male	6 (50.0)	9 (81.8)	15 (65.2)
Female	6 (50.0)	2 (18.2)	8 (34.8)
Race, *n* (%)			
White	4 (33.3)	9 (81.8)	13 (56.5)
Black or African American	3 (25.0)	1 (9.1)	4 (17.4)
Asian	2 (16.7)	0	2 (8.7)
American Indian or Alaska Native	0	0	0
Native Hawaiian or other Pacific Islander	0	0	0
Not reported	1 (8.3)	1 (9.1)	2 (8.7)
Unknown	2 (16.7)	0	2 (8.7)
Blood type, *n* (%)			
A	4/12 (33.3)	1/9 (11.1)	5 (23.8)
B	4/12 (33.3)	2/9 (22.2)	6 (28.6)
AB	0	1/9 (11.1)	1 (4.8)
O	4/12 (33.3)	5/9 (55.6)	9 (42.9)
Dialysis time, median years (min–max)	6.80 (2.9–12.9)	5.05 (0.2–24.2)	6.23 (0.2–24.2)
Waitlist time, median years (min–max)	6.0 (2.2–12.9)	3.6 (0.6–9.2)	5.3 (0.6–12.9)
Origin of renal insufficiency at study entry, *n* (%)			
Diabetes mellitus	1 (8.3)	0	1 (4.3)
Hypertension	0	1 (9.1)	1 (4.3)
Glomerular disease	2 (16.7)	2 (18.2)	4 (17.4)
ADPKD	0	1 (9.1)	1 (4.3)
Failure of previous transplant	6 (50.0)	4 (36.4)	10 (43.5)
Urologic disorders	0	2 (18.2)	2 (8.7)
Other	5 (41.7)	4 (36.4)	9 (39.1)
No. of prior kidney transplants, *n* (%)			
0	2 (16.7)	1 (9.1)	3 (13.0)
1	7 (58.3)	6 (54.5)	13 (56.5)
2	2 (16.7)	3 (27.3)	5 (21.7)
3	1 (8.3)	1 (9.1)	2 (8.7)
Prior sensitizing events at screening, *n* (%)	11 (91.7)	10 (90.9)	21 (91.3)
Pregnancy	1 (9.1)	0	1 (4.8)
Transfusion	6 (54.5)	3 (30.0)	9 (42.9)
Transplant	10 (90.9)	10 (100)	20 (95.2)
cPRA per central laboratory, median % (min–max)	99.99 (99.62–100.00)	99.95 (98.38–100.00)	99.97 (98.38–100.00)
cPRA per local laboratory with OPTN, median % (min–max)	99.99 (99.90–100.00)	99.42 (95.57–99.85)	99.90 (95.57–100.00)

ADPKD, autosomal dominant polycystic kidney disease; cPRA, calculated panel reactive antibody; OPTN, Organ Procurement and Transplantation Network.

The median cPRA per local laboratory assessment was 99.99% (99.90–100.00) and 99.42% (95.57–99.85) in cohorts A and B, respectively. When measuring cPRA by central laboratory, the median cPRA was 99.99% (99.62–100.00) and 99.95% (98.38–100.00), respectively.

All patients received a median of three cycles of isatuximab, with 12 weeks of exposure. The median relative dose intensity was 98.24% in cohort A and 98.38% in cohort B.

### Safety

Safety analysis showed any grade treatment-emergent AEs (TEAEs) occurred in 7 (30.4%) patients overall. No TEAEs were grade ≥3. One death occurred in cohort A due to disease complications not related to study treatment during site visit follow-up. A safety summary of TEAEs by AE preferred term can be seen in Table 2 and Supplemental Table 2. No treatment-emergent serious AEs were reported, and the only treatment-related AEs were infusion reactions.

**Table 2:** Safety summary of treatment-emergent adverse events by adverse event preferred term

No. (%)	All (*N*=23)	
	All Grades	Grade ≥3
Any event	7 (30.4)	0
Infusion reaction^a^	5 (21.7)	0
Nasopharyngitis	1 (4.3)	0
Headache	1 (4.3)	0
Tachycardia	1 (4.3)	0
Nasal congestion	1 (4.3)	0
Nausea	1 (4.3)	0
Myalgia	1 (4.3)	0
Temporomandibular joint syndrome	1 (4.3)	0
Chills	1 (4.3)	0
COVID-19	1 (4.3)	0

COVID-19, coronavirus disease 2019.

a Treatment-related.

At BL, anemia as laboratory abnormality occurred in 65.2%. Post-treatment, anemia occurrence increased to 82.6% of patients. At both BL and post-treatment, most occurrences were grade 1 in severity with no occurrence of grade ≥3. Lymphocytopenia as laboratory abnormality occurred in 25.0% of patients at BL, most of which were grade 1. Post-treatment, the occurrence of lymphocytopenia as laboratory abnormality was 56.3%, with most occurrences grade 1 and one grade 3 occurrence (6.3%). There were no instances of neutropenia during the trial. No patients had an on-treatment positive ADA response against isatuximab.

### PK of Isatuximab in Kidney Transplant Candidates

Isatuximab was quantifiable in plasma over the whole dosing period of 1 week after the first infusion at a dose of 10 mg/kg. The overall mean isatuximab maximum plasma concentration (C_max_) and area under the curve over 1 week (AUC_1 week_) were 290 *μ*g/ml and 24,700 *μ*g·h/ml, respectively, with moderate variability. A PK summary can be seen in Table 3 and Supplemental Table 3.

**Table 3:** Summary of pharmacokinetics of isatuximab after the first administration at a dose of 10 mg/kg

Mean±SD (CV %)	All (*N*=22)
C_max_, *μ*g/ml	290±109 (38)
*t*_max_^a^, h	3.46 (2.00−6.03)
AUC_1 week_, *μ*g·h/ml	24,700±7880 (32)^b^

AUC_1 week_, area under the curve over 1 week; C_max_, maximum plasma concentration; CV, coefficient of variation; *t*_max_, time to reach maximal concentration.

a Median (min−max), *t*_max_ was generally at end of infusion.

b *n*=20.

### Desensitization Activity in Highly Sensitized Kidney Transplant Candidates

The overall RR was 83.3% in cohort A and 81.8% in cohort B (Table 4). Median DoR was not reached in either cohort (cohort A 95% CI, 4.857 to not reached weeks; cohort B 95% CI, 4.143 to not reached weeks). Most responders had a decrease in the anti-HLA antibody level after treatment initiation which was maintained during the site visit follow-up period after stopping treatment. However, among all patients, there is minimal effect on the overall cPRA values. Only 39% of patients (4/12 and 5/11 in cohorts A and B, respectively) had reached target cPRA (*i.e.*, decrease in the cPRA level that would result in at least doubling the theoretical likelihood of finding a compatible donor).^19^ Approximately 47.8% (7/12 and 4/11 in cohorts A and B, respectively) had meaningful reduction in anti-HLA antibody titer, and 82.6% (10/12 and 9/11 in cohorts A and B, respectively) had at least one anti-HLA antibody with BL MFI ≥3000 reduced to <2000 (Table 4 and Supplemental Table 4). No BL clinical characteristics or laboratory features were observed to be associated with treatment response. Indeed, the small cohort sample size precludes any meaningful predictive biomarker analysis. In particular, given the polymorphic nature of the HLA system, as well as the variability in antibody strengths and other variables, a large cohort is required to enable more detailed analysis with high confidence.

**Table 4:** Summary of response rate in the efficacy-evaluable population on the basis of assigned cohort using screening calculated panel reactive antibody from local laboratory assessment

No. (%)	Cohort A (*n*=12)	Cohort B (*n*=11)	All (*N*=23)
No. of participants assessed	12	11	23
RR based on criterion 1	4 (33.3)	5 (45.5)	9 (39.1)
RR based on criterion 2	7 (58.3)	4 (36.4)	11 (47.8)
RR based on criterion 3	10 (83.3)	9 (81.8)	19 (82.6)
Overall RR	10 (83.3)	9 (81.8)	19 (82.6)
95% CI	51.6 to 97.9	48.2 to 97.7	61.2 to 95.0

CI, confidence interval; RR, response rate.

**Figure 2:**
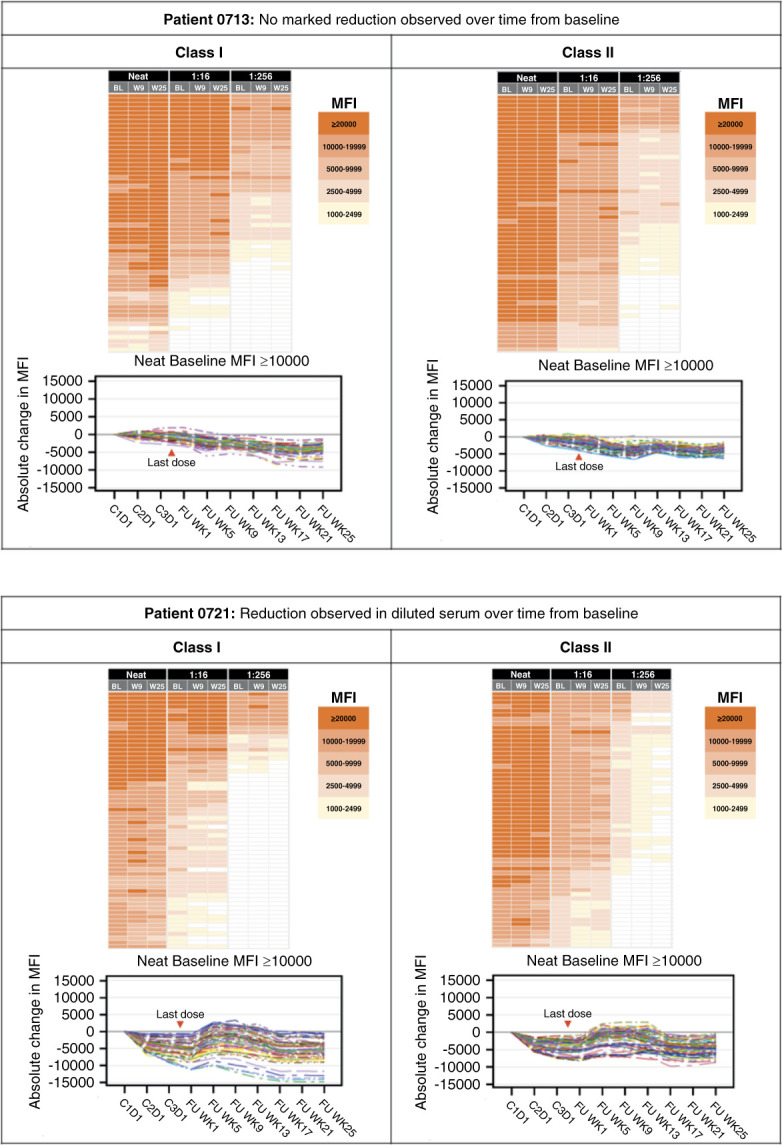
**Examples of patients who were responders per protocol but did not meet target cPRA in criterion 1.** Class I and class II anti-HLA antibody heat maps illustrate the MFI values of the top 60 HLA alleles (from the top, in descending order on the basis of their BL titer) at neat, 1:16, and 1:256 dilutions. Each dilution includes BL, W9 (approximately 9 weeks after the last dose), W25 (approximately 25 weeks after the last dose). Spaghetti plots illustrate the absolute change in MFIs in neat serum of anti-HLA antibodies with BL MFI ≥10,000. BL, baseline; C, cycle; cPRA, calculated panel reactive antibody; D, day; FUP, follow-up; MFI, mean fluorescence intensity; N, neat; WK, week.

cPRA alone is not sufficient to reflect a partial desensitization effect, and a composite end point that also included titer reduction and assessment of anti-HLA antibody profiles was therefore implemented to provide a better measurement. This is illustrated through the examples of partial responders, who met criterion 2 or 3 or both, but not criterion 1 (reaching target cPRA), as shown in Figure 2. Assessment of their titer and antibody profiles reveals more information on the desensitization effect of isatuximab. In some patients, although not considered responders based solely on cPRA, a marked and durable decrease in MFI, up to −15,000, was observed for some anti-HLA antibodies.

The example full profiles of responders, partial responders, and nonresponders are presented in Supplemental Figures 1–3. Of note, MFI values increased from BL over time in patient 0811, a nonresponder per protocol (Supplemental Figure 3C). This increase was observed from C2D1 onward, approximately 2.5 weeks after the patient received their second dose of coronavirus disease 2019 vaccine and at the time point where the highest stimulation of the immune system is expected to be observed. This potentially led to the nonspecific activation of dormant memory response to HLA.

### Transplant Outcomes

As of study cut-off date, a total of six patients treated with isatuximab received transplant offers (three each from cohorts A and B), all of which were from deceased donors. Four transplant offers were accepted. Reasons for declining an offer were offer not suitable for transplant and poor donor quality. Among the four patients who received transplant before study cut-off date, two of four were HLA incompatible with their donors before isatuximab treatment but were negative at the time of transplant. Three grafts were functioning with no report of rejection as of study cut-off date, while one graft in cohort A was lost due to thrombosis 1 day after transplant surgery with no reported rejection. As of February 2023, 9/11 patients from the two recruitment centers in Spain received kidney transplantation, five of whom received kidneys from previously incompatible donors.

### Pharmacodynamics and Immune Modulation of Isatuximab

**Figure 3:**
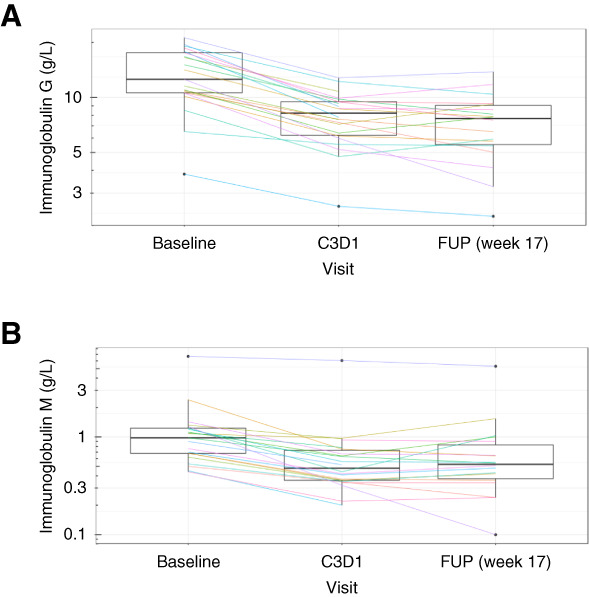
**Sustained decrease in total IgG and IgM levels up to last analyzed timepoint.** Evolution of (A) total IgG levels and (B) total IgM levels.

**Figure 4:**
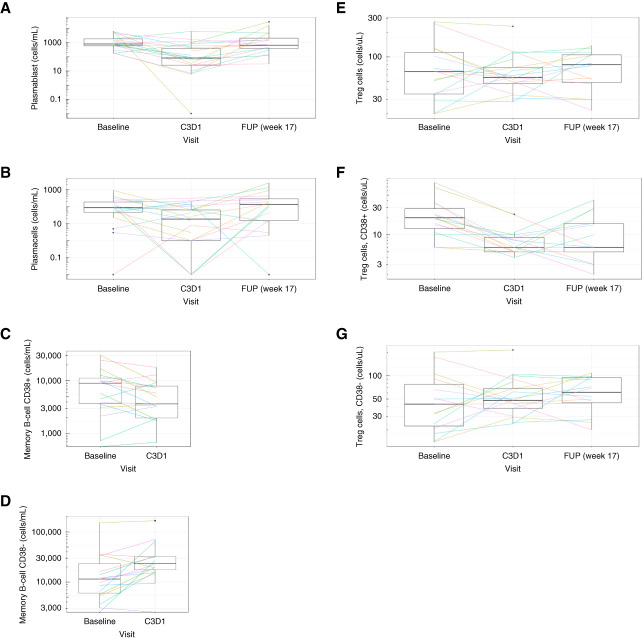
**No notable changes were observed in the total mBC or total Treg population, although decreases in plasmablasts and plasma cells were observed at C3D1 that returned to baseline by week 17 after last dose.** (A) Plasmablasts, (B) plasma cells, (C) CD38^+^ mBCs, (D) CD38^−^ mBCs, (E) overall Treg cells, (F) CD38^+^ Treg cells, and (G) CD38^−^ Treg cells over isatuximab treatment from BL to follow-up at week 17* *Plotted with logarithmic scale for ease of visualization. “0” values were replaced with “0.01.” mBC, memory B cell.

Using Ig levels as a surrogate, the results from the pharmacodynamics analysis support isatuximab target engagement. A sustained and significant decrease in total Ig levels was observed up to the last analyzed time point (*P* < 0.05, Supplemental Table 5), 17 weeks after last dose (IgG and IgM as shown in Figure 3, A and B). A decrease in peripheral plasmablasts (*P* = 0.025 and adjusted *P* value = 0.245) and plasma cells (*P* = 0.078 and adjusted *P* value = 0.706) was observed at C3D1 (week 9) compared with BL, with a trend of returning to BL at 17 weeks after the last dose (Figure 4, A and B). Although no notable change was observed in the total mBC population (data not shown), there was a decreasing trend in CD38^+^ switched mBCs (Figure 4C, *P* = 0.069) and an increase in CD38^−^ mBCs (Figure 4D, *P* = 0.006 and adjusted *P* value = 0.069). A significant decrease in NK cells was observed at C3D1 compared with BL (*P* < 0.001 and adjusted *P* value = 0.005), which is mainly driven by the depletion of CD38^+^ NK cells as no significant change was detected in the CD38^−^ NK cell population at all time points. No notable decreases in the total Treg population were observed, as a decrease in CD38^+^ Tregs was compensated by an increase of the CD38^−^ Treg compartments (Figure 4, E–G). No notable changes in other T-cell subset data were observed during the study follow-up (data not shown).

**Figure 5:**
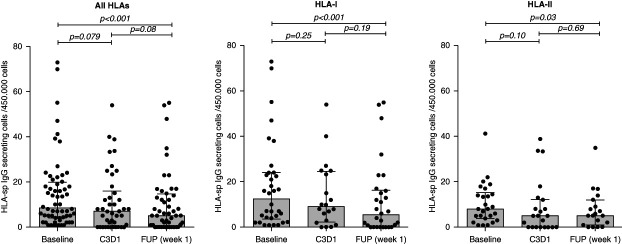
**Boxplots of mBC function.** Median IgG-secreting HLA-sp mBCs against all HLA antigens at distinct time points were 8.5 (4–19.75) at BL, 7 (0–15.7) at C3D1 and 5 (0.5–14.5) at FUP week 1, against class I HLA antigens were 12.5 (3.5–24) at BL, 9 (2.25–24.5) at C3D1, and 5.5 (0–16.3) at FUP week 1 and against class II HLA antigens were 8 (3.8–15.2) at BL, 5 (0–12.3) at C3D1, and 5 (1–12) at FUP week 1. ns, nonsignificant.

**Figure 6:**
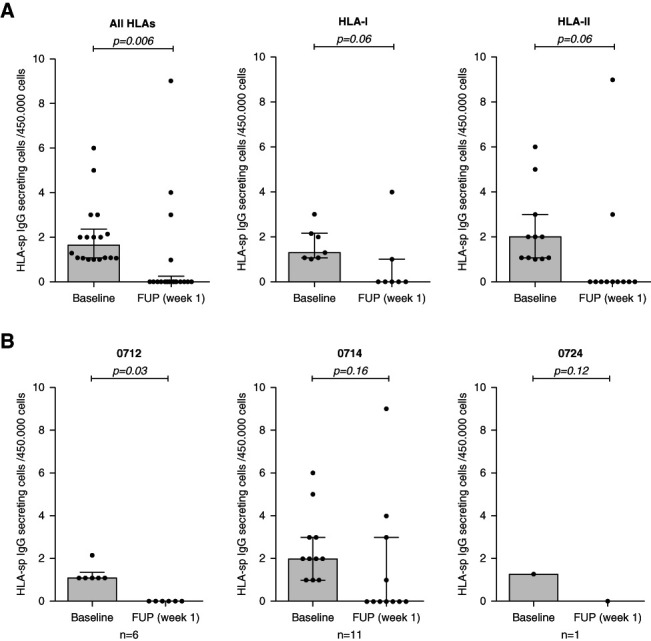
**Boxplots of HLA-specific IgG-producing bone marrow–residing plasma cells.** (A) Median IgG-secreting plasma cell frequencies at two distinct time points against all HLA antigens in the three tested patients were 1.6 (1.1–2.4) at BL and 0 (0–0.25) at FUP week 1, against HLA-I antigens were 1.3 (1.1–2.1) at BL and 0 (0–1) at FUP week 1, and against HLA-II antigens were 2 (1.1–3) at BL and 0 (0–0) at FUP week 1. (B) The median IgG-secreting plasma cell frequencies at BL and FUP week 1 in patient 0712 was 1.1 (1.1–1.3) versus 0 (0–0), in patient 0714 was 2 (1–3) versus 0 (0–3), and in patient 0724 was 1.28 versus 0.

The functional analyses of circulating HLA-specific mBCs revealed that treatment with isatuximab significantly reduced the frequencies of both class I and class II HLA-specific IgG-secreting mBCs, which was mostly achieved after receiving the total eight doses (Figure 5). Furthermore, a drastic reduction of HLA-specific IgG-producing bone marrow–residing plasma cells specific against classes I and II HLA antigens was also observed after three cycles of isatuximab therapy (Figure 6, A and B). Representative images of mBCs HLA-sp FluoroSpot before and after treatment are illustrated in Supplemental Figure 4. The analysis of the relationship between reduction of anti-HLA antibodies MFI from the sera and the respective HLA-sp mBC revealed that while 24.5% (12/49) of HLA antibody specificities were reduced in both compartments and 12% (6/49) were still concomitantly present both in the sera and by mBCs, up to 51% (25/49) of the Abs that were reduced in serum were unchanged in the functional in vitro assay by circulating mBC, harboring the same HLA specificity.

## Discussion

This study was designed to investigate whether the anti-CD38 therapy, isatuximab, has the potential to be an effective desensitization therapy, addressing a therapeutic gap left by currently available regimens. The study consisted of two cohorts, representing patients who may potentially benefit from desensitization.

Patients in cohort A represent the largest proportion of patients under the US KAS within the 100% cPRA category, with a significantly lower transplant rate and who are unlikely to undergo transplantation within a reasonable timeframe. Cohort B patients were those with cPRA 80.0%–99.89% who also receive prioritization allocation points. Despite this, the median cPRA in cohort B was similar to cohort A. BL characteristics also showed patients were on the waiting list for a kidney transplant for years (median 5.3 years, range 0.6–12.9) despite the recent advances in the field, demonstrating that there is still significant unmet clinical need under the current KAS.

Isatuximab monotherapy was well tolerated with a good safety profile in kidney transplant candidates, with a grade 1–2 infusion reaction rate of approximately 21%. No treatment-related infections were reported throughout the study, although the risk of hypogammaglobulinemia has been raised as a concern with anti-CD38 therapy due to CD38 being expressed in normal plasma cells, and as seen with the nonspecific elimination of IgG and IgM (Figure 2).^22,23^

A comprehensive population PK analysis in patients with relapsed/refractory multiple myeloma did not identify any effect of renal impairment on isatuximab PK.^24^ In this study, where renal function was even worse due to majority of patients being on dialysis, isatuximab PK exposure was comparable with those from other studies.^25–27^ Overall, these results complement the previous analyses mentioned above, showing no effect of renal impairment on isatuximab PK exposure. These results were expected as isatuximab is a monoclonal antibody, and thus a large molecule, and is eliminated by catabolism.

The data presented here support isatuximab's mechanism of action and achievement of target engagement *via* a sustained decrease in Ig levels. A decrease in peripheral CD38^+^ plasmablasts and plasma cells was observed, supported by a robust and sustained decrease of Ig levels after treatment was stopped for >17 weeks. In addition, HLA-specific IgG antibodies produced by circulating mBCs were also partially reduced, a finding that may account for the depleting effect of isatuximab to the mature class-switched mBC subset compartment expressing CD38. However, the peripheral cell population was small, and data should be interpreted cautiously. Notably, a significant reduction in HLA-specific IgG antibody production of bone marrow–residing plasma cells was observed in patients with evaluable bone marrow aspirates, highlighting the efficacy of isatuximab in targeting this central lymphoid compartment.

Prior publications investigating daratumumab hypothesized anti-CD38 treatment decreases Treg cells. In rhesus macaques with two sequential mismatched skin allografts desensitized with daratumumab and plerixafor before transplant, DSA levels were significantly reduced but this reduction was not maintained as all recipients showed a rapid rebound of antibodies, experienced T-cell–mediated rejection (TCMR), and developed rejection within 30 days of transplantation.^10^ Jordan *et al.* reported a case of a patient treated with daratumumab for standard-of-care resistant ABMR—ABMR resolved with minimal AEs, with significant reductions in circulating HLA class I and reductions in HLA class II, but the patient developed TCMR.^28^

In this study, no notable changes implicated in transplant rejection were observed in NK cells and T cells. Sufficient data are lacking to suggest whether the risk of TCMR was increased after treatment with isatuximab. However, no TCMR was observed in the transplanted patients treated with isatuximab as of study cut-off date. One patient transplanted approximately 15 months after the last dose of isatuximab (after study cut-off date) experienced acute rejection that was successfully treated with plasmapheresis and intravenous Ig, where the pathologic diagnosis includes mixed acute ABMR and TCMR due to the presence of interstitial infiltrate and tubulitis in addition to severe peritubular capillaritis with diffuse C4d staining.

Isatuximab demonstrated a durable decrease in anti-HLA antibodies, which appeared to be persistent during site visit follow-up period after stopping treatment (approximately 26 weeks after the last dose). Isatuximab also demonstrated partial desensitization activity by eliminating or lowering the titer of some antibodies, with minimal effect on the overall cPRA values. Nevertheless, Schinstock *et al.* show that a mild reduction in cPRA to 99.50%–99.89% may drastically increase the probability of transplant on the based on the current KAS.^29^ The minimal decrease in cPRA values is unsurprising as most broadly sensitized patients often have high titers of HLA antibody. As demonstrated in this study, desensitization activity cannot be reflected through cPRA values alone. Examining antibody titer reduction across the entire anti-HLA antibody profile of each patient provides a better assessment of desensitization efficacy. Therefore, a composite end point that included antibody elimination, titer reduction, and cPRA reduction proposed in this study may be more suitable for assessing desensitization therapies. The proposed criteria account for and minimize potential assay variability. For example, in criterion 3, only a reduction of antibodies to MFI <2000 from a BL of ≥3000 would be considered as antibody elimination on the basis of a potential 25% assay variability.^30^

Patients in this study received a limited number of doses of isatuximab and were subsequently followed up for 26 weeks on anti-HLA antibody level and immune cell profiling. The short treatment period and long follow-up duration design intended to explore the temporal mechanisms of antibody rebound if it occurred. A desired desensitization treatment regimen with isatuximab may then be adjusted based on the observed data. It is possible to administer isatuximab while the patient is on the waiting list for a deceased donor and start retreatment as needed to maintain their desensitized status. This may be as frequent as every 6 months on the basis of the available biomarker data indicating when plasma cells and plasmablasts start to return to BL levels. However, further study will be required to optimize the retreatment frequency.

Isatuximab may be further investigated as an option for adjunct therapy to existing desensitization therapy for patients on the kidney transplant waiting list to provide better durable responses due to its well-tolerated safety profile. For instance, isatuximab could be combined with a therapy that targets the mBC or germinal center B-cell compartments that do not express CD38, such as anti-CD20 antibody or belatacept, a fusion receptor protein that inhibits T-cell activation.^31^ As isatuximab targets plasma cells and does not target circulating anti-HLA antibodies, time is needed for the desensitization effect to be observed, considering the half-life of Ig. Combining isatuximab with an initial session of plasmapheresis or imlifidase may be an effective strategy to achieve fast and durable desensitization. However, these combinations need further clinical investigation, particularly to ascertain if there are adverse interactions with isatuximab. There is also potential for further investigation of the prevention or treatment of ABMR as an adjunct therapy on the basis of the durable plasma cell depletion and DSA suppression observed in this study.

This study is a biomarker-based mechanistic study investigating whether isatuximab monotherapy can durably decrease titers of anti-HLA antibody. Although there were patients transplanted during the study, these cannot be clearly attributed to isatuximab treatment, which needs to be tested in a randomized controlled study. However, whereas transplantability may seem to be a clinically meaningful measure of direct clinical benefit, there are intrinsic biases associated with patient selection, donor availability, different organ allocation policies across countries and regions, skill and aggressiveness of a transplant center, and difference in crossmatch positivity cutoff across transplant centers. It would also be challenging to test this hypothesis in a randomized controlled study in patients waiting for a deceased donor using transplant rate as the primary end point due to confounding factors, such as donor availability, varying criteria on donor crossmatch, and inconsistent use of desensitization regimens across transplant centers.

In summary, isatuximab was well tolerated in kidney transplant candidates, and monotherapy demonstrated a durable decrease in anti-HLA antibodies, with partial desensitization activity. The durable decrease in the anti-HLA antibody level observed was accompanied by reduction in alloantibody production sources (including long-lived plasma cells and in mBC function) after isatuximab treatment. The ultimate benefit of isatuximab as a monotherapy or as potential adjunct therapy in facilitating transplantation from previous incompatible donors will require corroboration from future controlled trials.

## Supplementary Material

## Data Availability

All data is included in the manuscript and/or supporting information.

## Supplemental Material

Supplemental Appendix 1. Desensitization response criteria.

Supplemental Table 1. HLA specificities assessed in circulating memory B cells and bone marrow plasma cells with the HLA-specific B-cell FluoroSpot in each patient.

Supplemental Table 2. Summary of safety by cohort.

Supplemental Table 3. Summary of pharmacokinetics of isatuximab by cohort after the first administration at a dose of 10 mg/kg.

Supplemental Table 4. Summary of number of anti-HLA antibody with baseline MFI ≥3000 reduced to <2000 as measured in a SAB assay (using central laboratory assessment).

Supplemental Table 5. Summary of pharmacodynamic changes from baseline to post-treatment.

Supplemental Figure 1. Examples of responder patients per protocol who met all three criteria.

Supplemental Figure 2. Examples of responder patients per protocol that did not meet target cPRA in criterion 1.

Supplemental Figure 3. Examples of nonresponder patients per protocol that met zero of three criteria.

Supplemental Figure 4. Representative images of mBCs and bone marrow plasma HLA-sp FluoroSpot before and after treatment.
