# Supplementary material for: Isatuximab Monotherapy for Desensitization in Highly Sensitized Patients Awaiting Kidney Transplant
Source: J Am Soc Nephrol. 2023 Dec 26;35(3):347–60. doi: 10.1681/ASN.0000000000000287 (PMC10914196; doi:10.1681/ASN.0000000000000287)

SUPPLEMENTAL FIGURES AND TABLES

Contents

Appendix 1. Desensitization Response Criteria…………………………………………………...2

Supplemental Table 1. HLA specificities assessed in circulating memory B cells and bone-marrow plasma cells with the HLA-specific B-cell Fluorospot in each patient…………………..4

Supplemental Table 2. Summary of safety by cohort……………………………………………..6

Supplemental Table 3. Summary of pharmacokinetics of isatuximab by cohort after the first administration at a dose of 10 mg/kg…………………………………………………………….7

Supplemental Table 4. Summary of number of anti-HLA-antibody with baseline MFI ≥3000 reduced to <2000 as measured in a SAB assay (using central laboratory assessment)……..8

Supplemental Table 5. Summary of pharmacodynamic changes from baseline to post-treatment ……………………..………………………………………………………………..9

Supplemental Figure 1. Examples of responder patients per protocol who met all 3 criteria…10

Supplemental Figure 2. Examples of responder patients per protocol that did not meet target cPRA in Criterion 1…………………………………………………………………………..12

Supplemental Figure 3. Examples of non-responder patients per protocol that met 0 of 3 criteria……………………………………………………………………………………...14

Supplemental Figure 4. Representative images of mBCs and bone-marrow plasma HLA-sp fluorospot before and after treatment……………………………………………………………..16

Appendix 1. Desensitization Response Criteria

The target cPRA (ie, reduction of cPRA required to achieve at least 100% increase in likelihood of compatible donor [LCD]) is calculated according to the following equation (Kransdorf EP et al. Hum Immunol 2017;78(3):252-6). Examples of target cPRA reduction are presented in Table A.

$$LCD = 1\ in \frac{1}{1 - cPRA}$$

Table A. Examples of target cPRA reduction.

Baseline		Target	
**cPRA**	**LCD**	**cPRA**	**LCD**
99.99%	1:10000	99.98%	1:5000
99.90%	1:1000	99.80%	1:500
99.80%	1:500	99.60%	1:250
99.60%	1:250	99.20%	1:125
99.50%	1:200	99.00%	1:100
99.00%	1:100	98.00%	1:50
97.50%	1:40	95.00%	1:20
95.00%	1:20	90.00%	1:10
90.00%	1:10	80.00%	1:5
80.00%	1:5	60.00%	1:2.5

Abbreviations: cPRA, calculated panel reactive antibodies; LCD, likelihood of compatible donor.

Participants with baseline cPRA 100.00% were assigned with cPRA 99.99% for computational purpose.

Antibody titer is defined as the last dilution of serum at which positive results is obtained (eg, MFI ≥2000) (Tambur AR, et al. Hum Immunol 2016;77(8):624-30). Examples of antibody titer based on MFI are illustrated in Table B below.

Table B. Examples of antibody titer.

	**Neat serum**	**1:2**	**1:4**	**1:8**	**1:16**	**1:32**	**Titer**
Antigen 1 MFI	13430	12492	6250	3123	1550	790	8
Antigen 2 MFI	18320	20820	10501	5206	2604	1302	16
Antigen 3 MFI	11020	5493	2750	1384	688	360	4

Abbreviations: MFI, mean fluorescence intensity.

Using similar concept, Table D illustrates an example in which the second predefined desensitization criterion (ie, ≥75% reduction in antibody titer from baseline to achieve target cPRA, see Table C) may be met. In this example, participant's baseline cPRA is measured by SAB as 97.50%, the target cPRA is therefore 95.00% as defined in Table A. Through serial dilution of the serum collected at baseline, titer required to achieve target cPRA is 16 (ie, achieved at 1:16 dilution). Subsequent post-treatment serum is therefore diluted at 1:4 (ie, 75% reduction from 1:16) to determine whether target cPRA is achieved. At Cycle 3 Day 1, target cPRA is reached at 1:4 dilution therefore meeting the aforementioned predefined desensitization criterion.

Table C. 75% reduction in antibody titer from baseline

**Baseline**		**75% reduction**	
**Titer**	**Dilution**	**Titer**	**Dilution**
2	1:2	1	Neat
4	1:4	1	Neat
8	1:8	2	1:2
16	1:16	4	1:4
32	1:32	8	1:8
64	1:64	16	1:16
128	1:128	32	1:32
256	1:256	64	1:64
512	1:512	128	1:128

Table D. Example of ≥75% reduction in antibody titer from baseline to achieve target cPRA

	**Neat serum**	**1:2 Titer=2**	**1:4 Titer=4**	**1:8 Titer=8**	**1:16 Titer=16**	**Response criterion met**
Baseline cPRA	97.50%	97.50%	97.50%	96.00%	95.00% (target cPRA)	
C2D1 cPRA	96.00%		96.00%			No
C3D1 cPRA	96.00%		95.00% (target cPRA)			Yes

Abbreviations: C2D1, cycle 2 day 1; C3D1, cycle 3 day 1; cPRA, calculated panel reactive antibodies.

Supplemental Table 1. HLA specificities assessed in circulating memory B cells and bone-marrow plasma cells with the HLA-specific B-cell Fluorospot in each patient

**Patient ID**	**HLA specificities**	**Patient ID**	**HLA specificities**
**Circulating HLA-specific memory B cells**		**Bone marrow-residing HLA-specific Plasma cells**	
712	A*23:01	712	A*24:02
712	A*24:02	712	A*24:03
713	A*03:01	712	A*34:02
713	A*11:01	712	DRB1*14:01
713	B*07:02	712	DRB3*02:02
713	DRB1*01:03	712	DRB3*03:01
713	DRB1*11:04	714	A*68:02
714	A*02:01	714	B*37:01
714	B*57:01	714	B*67:01
714	DRB1*01:01	714	DQB1*03:02 DQA1*02:01
714	DRB1*04:02	714	DRB1*01:02
714	DRB1*07:01	714	DRB1*04:01
714	DRB1*10:01	714	DRB1*07:01
714	DRB1*11:01	714	DRB1*10:01
716	A*36:01	714	DRB1*15:01
717	A*01:01	714	DRB4*01:03
717	A*03:01	714	DRB5*02:02
717	A*24:02	724	B*27:05
717	A*29:02		
717	A*80:01		
717	B*07:02		
717	B*15:03		
717	B*15:12		
717	B*44:02		
717	B*45:01		
717	B*55:01		
717	DQB1*05:02/DQA1*01:02		
717	DQB1*06:03/DQA1*01:03		
717	DQB1*02:01/DQA1*02:01		
717	DQB1*02:02/DQA1*02:01		

717	DQB1*04:01/DQA1*02:01
717	DQB1*02:01/DQA1*03:01
717	DQB1*04:02/DQA1*04:01
718	A*01:01
718	A*23:01
718	A*24:02
718	A*36:01
718	B*57:01
718	DRB1*10:01
718	DRB1*15:03
719	A*01:01
719	A*36:01
719	DQB1*03:02/DQA1*02:01
719	DQB1*04:01/DQA1*02:01
719	DQB1*03:01/DQA1*06:01
722	A*23:01
722	B*07:02
722	B*51:01
722	DQB1*05:02/DQA1*01:02
722	DQB1*06:02/DQA1*01:02
723	DRB1*04:04
723	DRB3*03:01
724	A*02:01
724	A*11:01
724	A*68:01
724	B*27:05
724	B*37:01
724	DQB1*02:01 /DQA1*02:01
724	DQB1*02:01 /DQA1*03:01
724	DRB3*02:02

Supplemental Table 2. Summary of safety by cohort

n (%)	Cohort A (n=12)		Cohort B (n=11)	
	All grades	Grade ≥3	All grades	Grade ≥3
Any event	3 (25.0)	0	4 (36.4)	0
Infusion-related reaction	2 (16.7)	0	3 (27.3)	0
Nasopharyngitis	1 (8.3)	0	0	0
Headache	1 (8.3)	0	0	0
Tachycardia	1 (8.3)	0	0	0
Nasal congestion	1 (8.3)	0	0	0
Nausea	1 (8.3)	0	0	0
Myalgia	1 (8.3)	0	0	0
Temporomandibular Joint Syndrome	1 (8.3)	0	0	0
Chills	1 (8.3)	0	0	0
COVID-19	0	0	1 (9.1)	0

COVID-19, coronavirus disease.

Supplemental Table 3. Summary of pharmacokinetics of isatuximab by cohort after the first administration at a dose of 10 mg/kg

Mean ± SD [CV%]	Cohort A (n=11)	Cohort B (n=11)
C_{max} (µg/mL)	295 ± 128 [43]	285 ± 94 [33]
t_{max}* (h)	3.67 (2.00−6.03)	3.40 (2.25−4.63)
$AUC_{1\ week}$ (µg•h/mL)	29400 ± 7400 [25]†	20000 ± 5240 [26]†

*Median (min−max), t_{max} was generally at end of infusion

†n=10

$AUC_{1\ week}$, area under the curve over 1 week; C_{max}, maximum plasma concentration; CV, coefficient of variation; SD, standard deviation; t_{max}, time to reach maximal concentration

Supplemental Table 4. Summary of number of anti-HLA-antibody with baseline MFI ≥3000 reduced to <2000 as measured in a SAB assay (using central laboratory assessment)

	**Cohort A (n=12)**	**Cohort B (n=11)**	**All (N=23)**
Maximum number of anti-HLA-antibody reduced [n (%)]			
Number of participants assessed	12	11	23
None	2 (16.7)	2 (18.2)	4 (17.4)
1-5	4 (33.3)	4 (36.4)	8 (34.8)
>5-10	4 (33.3)	4 (36.4)	8 (34.8)
>10-15	1 (8.3)	0	1 (4.3)
>15	1 (8.3)	1 (9.1)	2 (8.7)
Baseline MFI 3000<6000			
None	2 (16.7)	2 (18.2)	4 (17.4)
1-5	5 (41.7)	5 (45.5)	10 (43.5)
>5-10	3 (25.0)	3 (27.3)	6 (26.1)
>10-15	1 (8.3)	0	1 (4.3)
>15	1 (8.3)	1 (9.1)	2 (8.7)
Baseline MFI 6000<10000			
None	9 (75.0)	9 (81.8)	18 (78.3)
1-5	3 (25.0)	1 (9.1)	4 (17.4)
>5-10	0	1 (9.1)	1 (4.3)
>10-15	0	0	0
>15	0	0	0
Baseline MFI ≥10000			
None	12 (100)	11 (100)	23 (100)

Supplemental Table 5. Summary of statistical testing in pharmacodynamic changes from baseline to post-treatment

Biomarker	C3D1	C3D1 (p-value)	C3D1 (adjusted p-value)	FUP (Week 17)	FUP Week 17 (p-value)	FUP (Week 17) adjusted p-value
Treg cells (cells/µL)	13	0.889	1.000	10	0.557	1.000
Treg cells, CD38+ (cells/µL)	13	0.004	0.046	10	0.221	1.000
Treg cells, CD38- (cells/µL)	13	0.376	1.000	10	0.275	1.000
Plasma cells (cells/mL)	14	0.078	0.706	12	0.380	1.000
Plasmablasts (cells/mL)	14	0.025	0.245	12	0.791	1.000
Immunoglobulin G (g/L	20	<0.001	<0.001	16	<0.001	<0.001
Immunoglobulin M (g/L)	20	<0.001	0.001	16	0.002	0.022
NK cells, CD38- (cells/µL)	13	0.085	1.000	10	0.200	1.000
NK cells, CD38+ (cells/µL)	13	<0.001	0.005	10	0.232	1.000
NK cells (cells/µL)	13	<0.001	0.005	10	0.275	1.000
Memory B-cell CD38+ (cells/mL)	16	0.083	0.706	N/A	N/A	N/A
Memory B-cell CD38- (cells/mL)	16	0.006	0.069	N/A	N/A	N/A

Supplemental Figure 1. Examples of responder patients per protocol who met all 3 criteria; (A) Antibody titration heat map, Plots of MFI change in anti-HLA antibody levels for antibodies with (B) baseline MFI of 3000 to <1000, and (C) baseline MFI ≥10000

A.

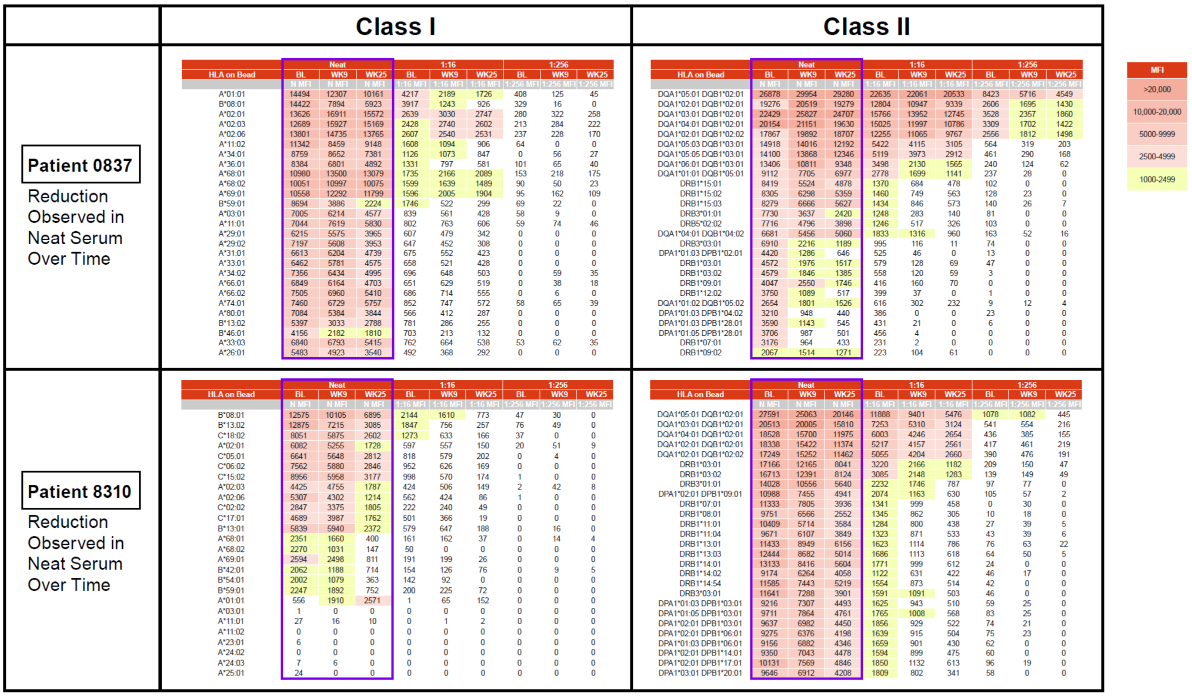

B.

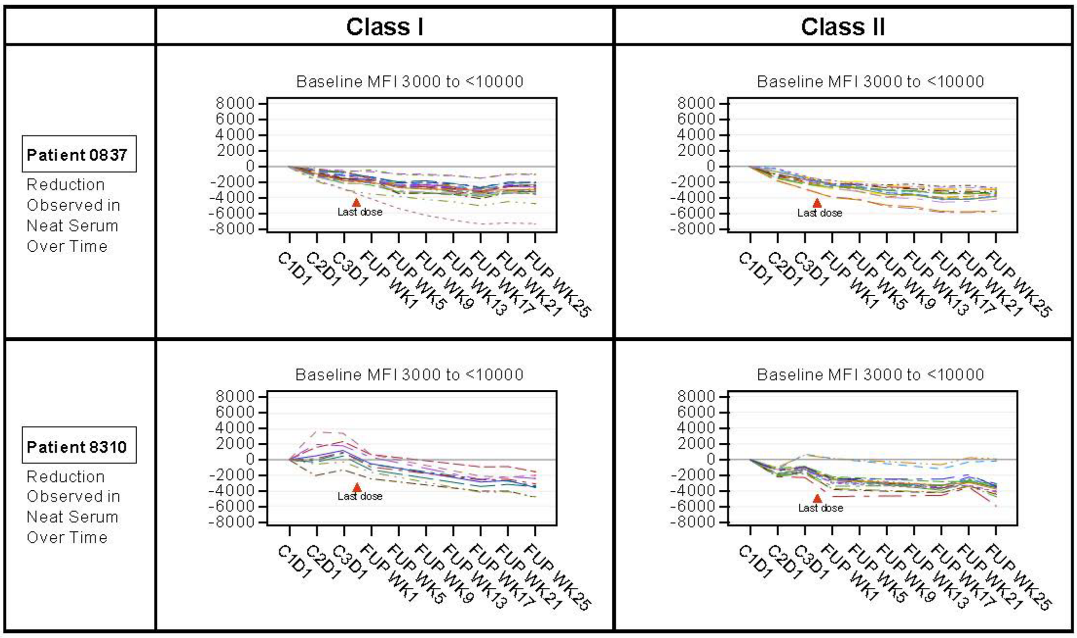

C.

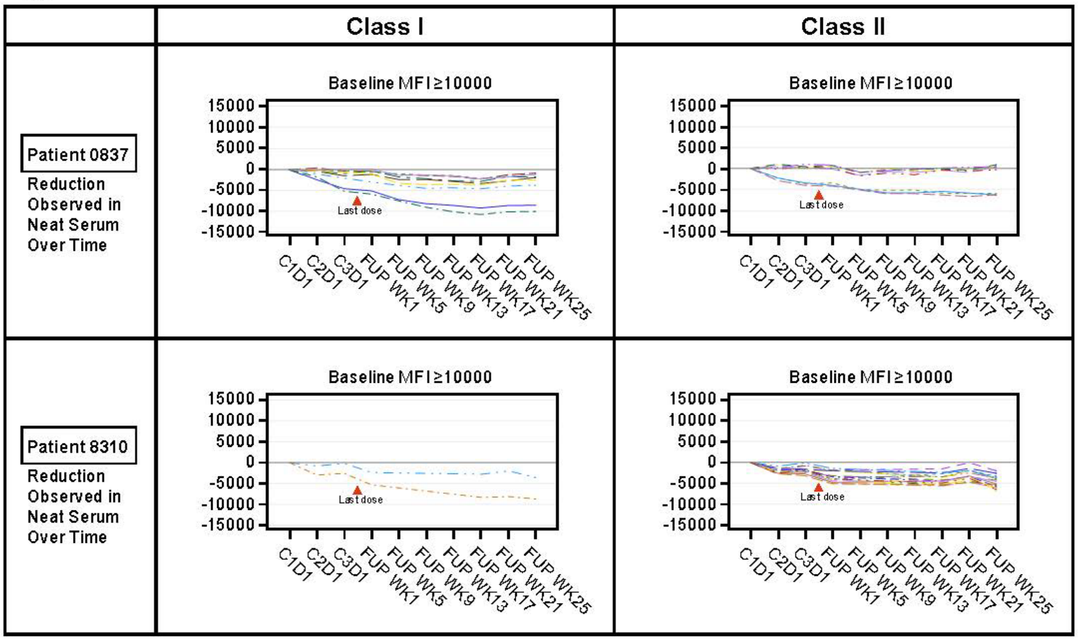

C, cycle; D, day; FUP, follow-up; HLA, human leukocyte antigen; MFI, mean fluorescence intensity; N, neat; WK, week.

Supplemental Figure 2. Examples of responder patients per protocol that did not meet target cPRA in Criterion 1; (A) Antibody titration heat map, (B and C) plots of MFI change in anti-HLA antibody levels for (B) antibodies with baseline MFI of 3000 to <1000, and (C) baseline MFI ≥10000

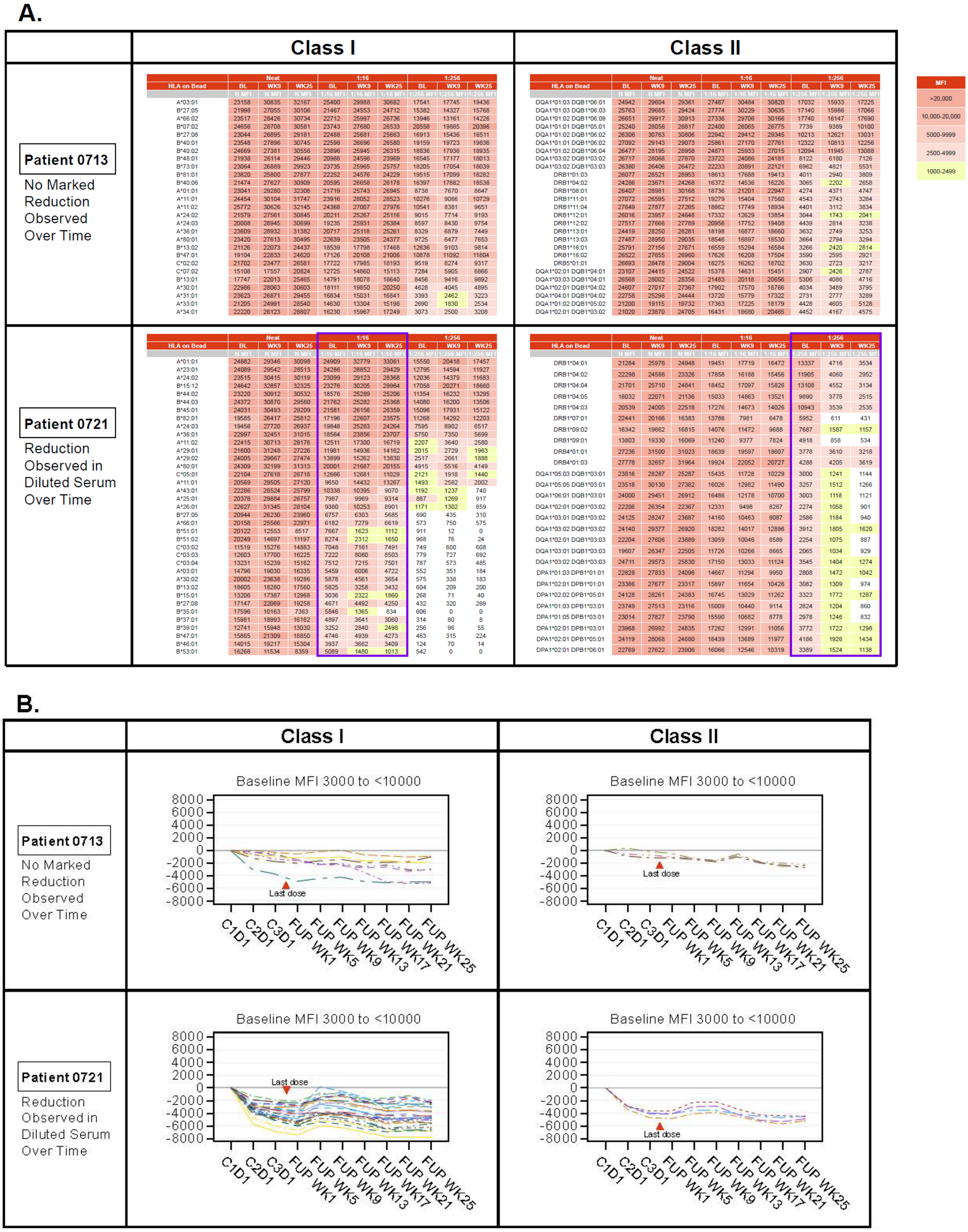

C.

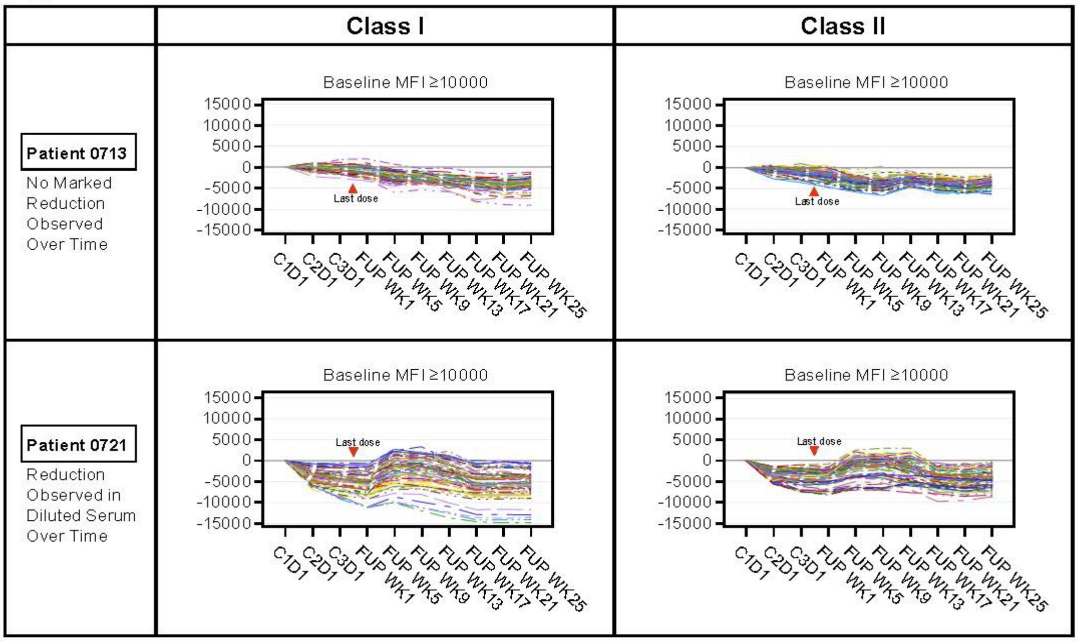

C, cycle; D, day; FUP, follow-up; HLA, human leukocyte antigen; MFI, mean fluorescence intensity; WK, week.

Supplemental Figure 3. Examples of non-responder patients per protocol that met 0 of 3 criteria; (A) Antibody titration heat map and (B and C) plots of MFI change in anti-HLA antibody levels for antibodies with (B) baseline MFI of 3000 to <1000, and (C) baseline MFI ≥10000

A.

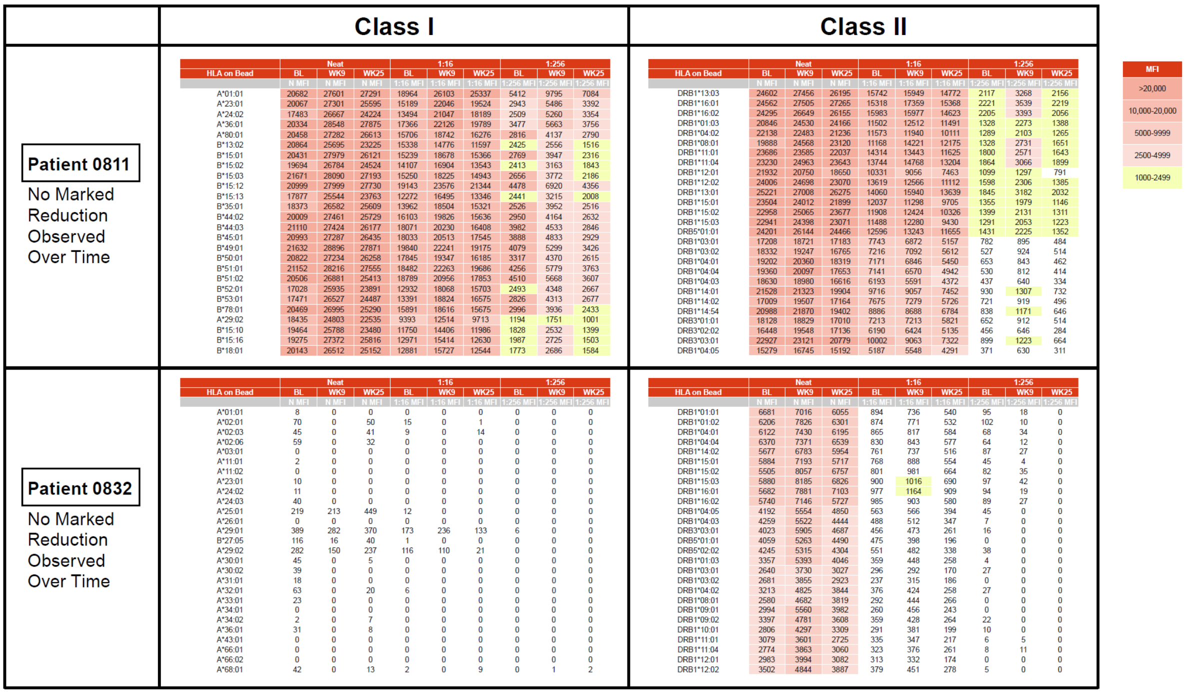

B.

	Class I	Class II
**Patient 0811**<br>No Marked Reduction Observed Over Time	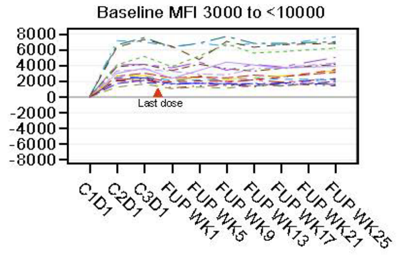	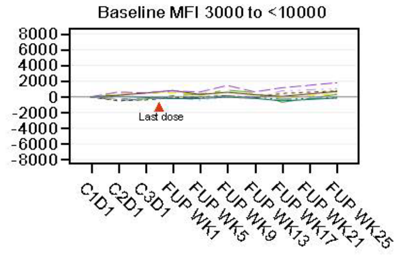
**Patient 0832**<br>No Marked Reduction Observed Over Time	No detectable Class I anti-HLA antibody at baseline MFI 3000 to <10000	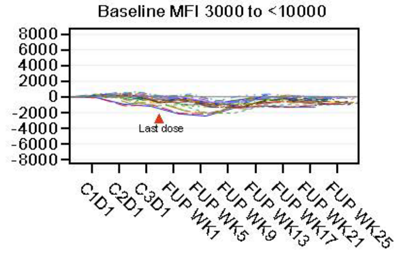

C.

	Class I	Class II
**Patient 0811**<br>No Marked Reduction Observed Over Time	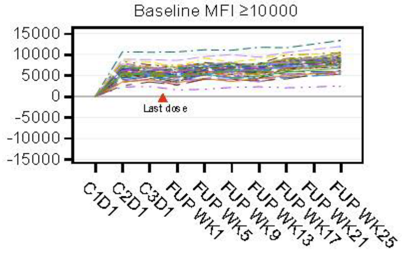	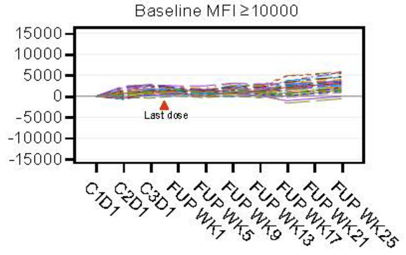
**Patient 0832**<br>No Marked Reduction Observed Over Time	No detectable Class I anti-HLA antibody at baseline MFI ≥10000	No detectable Class II anti-HLA antibody at baseline MFI ≥10000

C, cycle; D, day; FUP, follow-up; HLA, human leukocyte antigen; MFI, mean fluorescence intensity; WK, week.

Supplemental Figure 4. Representative images of mBCs HLA-sp B-cell fluorospot before and after treatment.

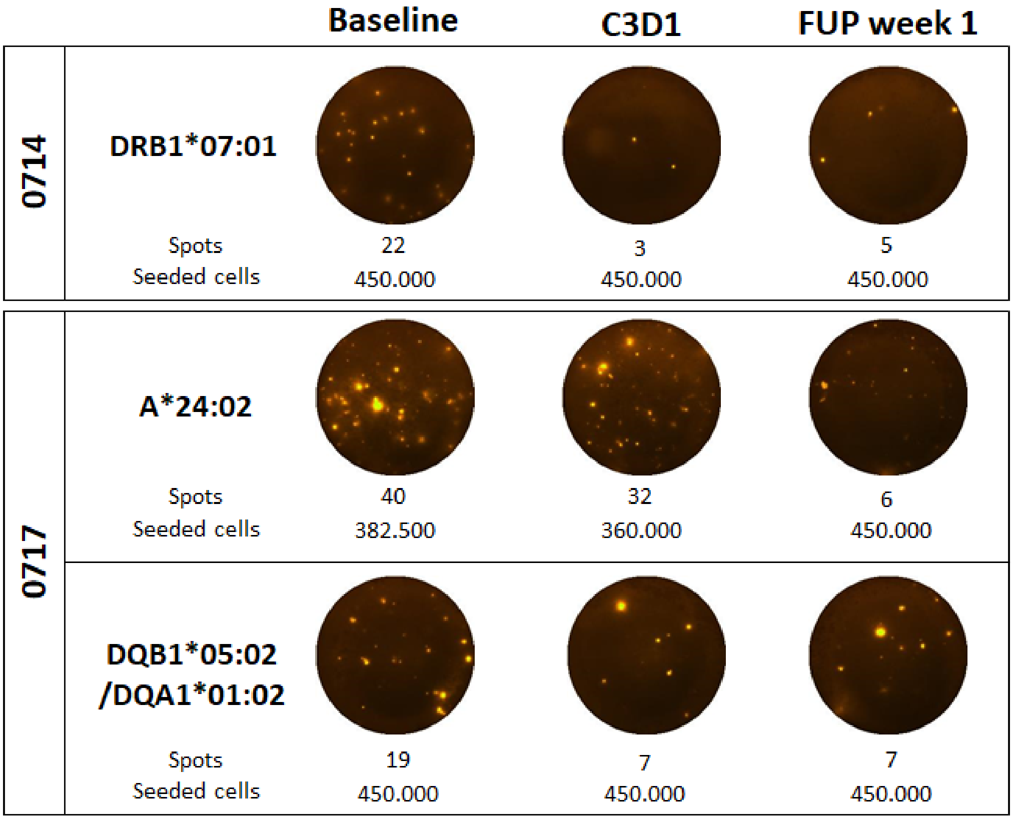

Supplement: Supplementary file 1 [file jasn-35-347-s001.pdf]
